# Motor Cortex Excitability and BDNF Levels in Chronic Musculoskeletal Pain According to Structural Pathology

**DOI:** 10.3389/fnhum.2016.00357

**Published:** 2016-07-15

**Authors:** Wolnei Caumo, Alícia Deitos, Sandra Carvalho, Jorge Leite, Fabiana Carvalho, Jairo Alberto Dussán-Sarria, Maria da Graça Lopes Tarragó, Andressa Souza, Iraci Lucena da Silva Torres, Felipe Fregni

**Affiliations:** ^1^Post-graduate Program in Medical Sciences, School of Medicine, Universidade Federal do Rio Grande do Sul (UFRGS)Porto Alegre, Brazil; ^2^Laboratory of Pain and Neuromodulation at UFRGSPorto Alegre, Brazil; ^3^Anesthesiologist, Pain and Palliative Care Service at Hospital de Clínicas de Porto Alegre (HCPA)Porto Alegre, Brazil; ^4^Pain and Anesthesia in Surgery Department, School of Medicine, UFRGSPorto Alegre, Brazil; ^5^Neuropsychophysiology Laboratory, CIPsi, School of Psychology (EPsi), University of Minho, Campus de GualtarBraga, Portugal; ^6^Post-graduate Program in Health and Human Development, La Salle University CenterCanoas, Brazil; ^7^Department of Pharmacology, Instituto de Ciências Básicas da Saúde, UFRGSPorto Alegre, Brazil; ^8^Berenson-Allen Center for Noninvasive Brain Stimulation, Department of Neurology, Beth Israel Deaconess Medical Center, Harvard Medical SchoolBoston, MA, USA

**Keywords:** short intracortical inhibition, brain-derived neurotrophic factor, central sensitization, conditioned pain modulation, osteoarthritis, fibromyalgia, myofascial pain syndrome

## Abstract

The central sensitization syndrome (CSS) encompasses disorders with overlapping symptoms in a structural pathology spectrum ranging from persistent nociception [e.g., osteoarthritis (OA)] to an absence of tissue injuries such as the one presented in fibromyalgia (FM) and myofascial pain syndrome (MPS). First, we hypothesized that these syndromes present differences in their cortical excitability parameters assessed by transcranial magnetic stimulation (TMS), namely motor evoked potential (MEP), cortical silent period (CSP), short intracortical inhibition (SICI) and short intracortical facilitation (SICF). Second, considering that the presence of tissue injury could be detected by serum neurotrophins, we hypothesized that the spectrum of structural pathology (i.e., from persistent nociception like in OA, to the absence of tissue injury like in FM and MPS), could be detected by differential efficiency of their descending pain inhibitory system, as assessed by the conditioned pain modulation (CPM) paradigm. Third, we explored whether brain-derived neurotrophic factor (BDNF) had an influence on the relationship between motor cortex excitability and structural pathology. This cross-sectional study pooled baseline data from three randomized clinical trials. We included females (*n* = 114), aged 19–65 years old with disability by chronic pain syndromes (CPS): FM (*n* = 19), MPS (*n* = 54), OA (*n* = 27) and healthy subjects (*n* = 14). We assessed the serum BDNF, the motor cortex excitability by parameters the TMS measures and the change on numerical pain scale [NPS (0–10)] during CPM-task. The adjusted mean (SD) on the SICI observed in the absence of tissue injury was 56.36% lower than with persistent nociceptive input [0.31(0.18) vs. 0.55 (0.32)], respectively. The BDNF was inversely correlated with the SICI and with the change on NPS (0–10)during CPM-task. These findings suggest greater disinhibition in the motor cortex and the descending pain inhibitory system in FM and MPS than in OA and healthy subjects. Likewise, the inter-hemispheric disinhibition as well as the dysfunction in the descending pain modulatory system is higher in chronic pain without tissue injury compared to a structural lesion. In addition, they suggest that a greater level of serum BDNF may be involved in the processes that mediate the disinhibition of motor cortex excitability, as well as the function of descending inhibitory pain modulation system, independently of the physiopathology mechanism of musculoskeletal pain syndromes.

## Introduction

A central sensitivity syndrome (CSS) is a cluster of symptoms including psychological distress, sleep disturbances, fatigue, pain, allodynia, hyperalgesia, and expansion of the receptive field (Yunus, 2007, 2008), which overlap with many chronic pain disorders. Despite the substantial overlapping, there is no consensus on the presence of these symptoms and structural pathology. For instance, in fibromyalgia (FM), chronic tensional headache and myofascial pain syndrome (MPS) the evidence of structural pathology is scarce. In contrast, in other conditions like osteoarthritis (OA), there is strong evidence of anatomic structural pathology that accounts for persistent nociceptive input. Nonetheless, irrespective of the amount of visible injury, these chronic pain conditions share a cluster of symptoms that support the hypothesis of the presence of central sensitization (CS) phenomenon. This neuronal event comprises an abnormal state of responsiveness for nociceptor stimuli. Thus, the pain arises as a consequence of changes within the central nervous system (CNS) that amplifies the response to nociceptive inputs across many organ systems and fails to suppress noise signals (Woolf and Salter, 2000; Ji et al., 2003).

At the cellular level, the CS comprises an impaired function of neurons and circuits in nociceptive pathways, in which exist increased membrane excitability, synaptic efficacy, or reduced inhibition (Latremoliere and Woolf, 2009). Sensitized neurons of the spinal dorsal horn exhibit increased spontaneous activity and response to subthreshold stimulation, reduction in the threshold for activation, and an enlargement of their receptive fields (Latremoliere and Woolf, 2009).

Thus, CSS pain arises from different abnormal mechanisms, including increased presynaptic release of excitatory neurotransmitters that will, in turn, elicit a greater postsynaptic response, by increasing the excitability of the postsynaptic membrane (Woolf et al., 1994; Craig, 2003). Then, changes in the microglia, astrocytes, gap junctions and gene transcription, contribute to the maintenance of this general state of excitation. Moreover, as part of the spinal microglial activation, the brain-derived neurotrophic factor (BDNF) is released, further contributing to the induction and maintenance of the CS (Trang et al., 2011).

Besides clinical complaints, the CSS also shares pathophysiological mechanisms. In OA, lower pain threshold and punctual hyperalgesia has been shown in areas of referred pain rather than on the original area of tissue injury (O’Driscoll and Jayson, 1975; Bajaj et al., 2001), which has been thought to reflect brainstem activation, as shown by functional magnetic resonance imaging (fMRI; Gwilym et al., 2009). In FM, the CS has been associated with the widespread reduction in thermal and mechanical pain thresholds (Gibson et al., 1994), inducing temporal summation, muscular hyperalgesia and pain, which are attenuated experimentally with the use of ketamine (Graven-Nielsen et al., 2000). Similarly, the persistent muscular tension experienced in the MPS has been hypothesized to induce CS (Fernández-de-las-Peñas et al., 2009), which could progressively produce the lead changes in the SNC. Although the BDNF is a common key player in the CS process, our research team recently showed that its serum levels might differ among chronic pain syndromes (CPS) (Deitos et al., 2015). In fact, the BDNF is a neurotrophic factor capable of strengthening glutamatergic synapses, while it weakens GABAergic synapses. The increase of this neurotrophic factor inverts the polarity of GABA currents in dorsal horn neurons (Coull et al., 2005). Thereby the GABAergic system loses the capacity to downregulate of the Cl-cotransporter K^+^-Cl^−^ exporter (KCC2) expression in the dorsal horn (Rivera et al., 2002; Zhang et al., 2008). Thus, the accumulation intracellular of Cl^−^ limits the GABAergic inhibitory effect on these nociceptors, thereby promoting the disinhibition (Latremoliere and Woolf, 2009), which results in a persistent and amplified response to nociceptive inputs and fails to suppress noise signals (Woolf and Salter, 2000; Ji et al., 2003). Although the cross-talk among BDNF and chronic pain is a complex phenomenon, and the underlying mechanisms responsible for such observations remain poorly understood, the differential BDNF levels might be utility in distinguishing CS syndromes with and without structural pathology (Deitos et al., 2015).

Considering that the CSS is the utmost clinical picture of dysfunctional neuronal circuits where the defective inhibitory function stands out, it is reasonable to consider the use of neuronal inhibition indexes to increase our mechanistically knowledge about the underlying neural substrates of CSS. Fortunately, it is nowadays possible to clinically assess the motor intracortical inhibition (ICI) and probing neural plasticity (Schwenkreis et al., 2011) using the motor cortex excitability by transcranial magnetic stimulation (TMS) paradigms. Accordingly, in the neuropathic pain compared to healthy controls (HCs) the repetitive transcranial magnetic stimulation (rTMS) improved the cortical disinhibition indexed by the ICI and by a shorter cortical silent period (CSP; Lefaucheur et al., 2006). Similar results were observed in other chronic pain conditions, such as in FM (Salerno et al., 2000) and complex regional pain (Schwenkreis et al., 2003). While in healthy subjects’ experimental pain using capsaicin, the rTMS applied over the dorsolateral prefrontal cortex (DLPFC) decreased the short intracortical inhibition (SICI; Fierro et al., 2010) and induced a significant anti-nociceptive effect in the capsaicin pain model (Brighina et al., 2011). Thereby, this set of findings suggest that the TMS, permit us modulate and also assess the state of the balance of excitatory and inhibitory system involved in the physiopathology of the CSS, which is a fundamental process to develop and maintain the CPS.

Therefore, the present study aims to explore tools to assess clinically some of the mechanisms likely associated with the CS. We evaluated the cortical excitability, the function in the descending pain modulatory system, and their relationships with the BDNF in three CS syndromes of chronic pain with different pieces of evidence of tissue injury: OA, FM, and MPS. Thus, we explored three hypotheses. First, we hypothesized that these syndromes present differences in their cortical excitability parameters assessed by TMS, namely motor evoked potential (MEP), CSP, SICI and short intracortical facilitation (SICF). Second, considering that the CS in the absence of tissue injury could be detected by serum neurotrophins, we hypothesized that the spectrum of structural pathology (i.e., from persistent nociception like in OA, to the absence of tissue injury like in FM and MPS), could be detected by differential efficiency of their descending pain inhibitory system, as assessed by the conditioned pain modulation (CPM) paradigm. Third, we explored whether BDNF had an influence on the relationship between motor cortex excitability and structural pathology.

## Materials and Methods

### Design Overview, Settings, and Participants

**Figure 1:**
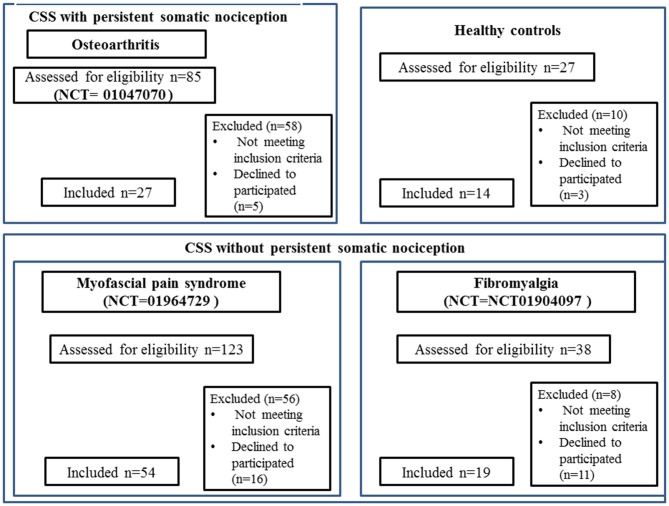
**CONSORT 2010 flow diagrams**. The present cross-sectional study pooled baseline data from three clinical trials run at Hospital de Clínicas de Porto Alegre (HCPA), and that recruited women with fibromyalgia (FM; NCT01904097), myofascial pain syndrome (MPS; NCT01964729), osteoarthritis (OA; NCT01747070), and CSS indicates central sensitivity syndrome; VAS, visual analog scale.

This protocol was approved by the Institutional Ethics Committee at the Hospital de Clínicas de Porto Alegre (HCPA; application no. 1005-55, Post-Graduate Research Group). All of the trials ran their respective protocols with the approval of the HCPA Ethics Committee and obtained written informed consent from all subjects. We conducted a cross-sectional study pooling baseline data from three clinical trials. The sample involved women with chronic pain conditions associated with CS syndromes but without evidence of structural pathology (FM NCT01904097 and MPS NCT01964729), and chronic pain conditions with CS symptoms due to known organic pathology (i.e., OA NCT01747070). Details of the inclusion of each study can be seen in Figure 1. All subjects were recruited by directly contacting them from the institutional chronic pain clinic, by referrals from other clinic units, and through media advertising. Besides their particular criteria, all trials excluded subjects who failed to understand Brazilian Portuguese.

The baseline data from the samples of three experiments conducted in our institution were retrieved. All the studies used the 100 mm visual analog scale (VAS) for pain, ranging from no pain (0 mm) to the worst pain imaginable (100 mm). Only subjects that reported pain equal or higher than 40 mm in the VAS [i.e., moderate or severe pain (Palos et al., 2006)] for more than 3 months, and that were associated with functional disability, were included in this study. Functional disability was assessed by a structured questionnaire containing dichotomous questions (yes/no) about how the chronic syndrome had interfered with their activities in the past 3 months, namely with: (1) work; (2) responsibilities at home; (3) enjoyable activities; (4) relationships; (5) personal goals; and (6) thinking clearly, problem-solving, concentrating, or recall. To be included, patients had to have at least one affirmative answer (i.e., Yes) to the questionnaire. Additionally, the Western Ontario and McMaster Universities Osteoarthritis Index (WOMAC) was used in the OA trial, as it is a reliable, and sensitive instrument commonly used to assess pain and disability in other studies of knee OA (Nunes et al., 2013). Only OA subjects with a disability score on the WOMAC were included.

Each clinical trial had rigorous inclusion criteria, and the diagnoses were confirmed by a physician with over 15 years of experience in chronic pain conditions. Specifically, the diagnosis was determined by the presence of clinical complaints, current and past medication, medical and psychiatric history, and current medical and psychiatric diagnosis. Trials criteria are summarized as follows:

1. Diagnosis criteria for chronic MPS included regional dull, achy, or deep pain with normal neurological examination; the presence of trigger or tender points, taut bands, palpable nodules; and exacerbation by stress, which could involve decreased range of motion and ropiness in the muscle (Tough et al., 2007). To distinguish neuropathic pain from ongoing nociception, the Neuropathic Pain Diagnostic Questionnaire (DN4) was applied to all subjects. Only those with a neuropathic component (score Z4) were included to standardize the severity of MPS.
2. FM diagnosis adhered to 2010 American College of Rheumatology criteria (Wolfe et al., 2011).
3. OA required the clinical and radiographic criteria of the American College of Rheumatology.
4. Pain-free control volunteers were invited using media advertisement and were prospectively recruited. We used a standard screening questionnaire to assess if they fulfilled the inclusion criteria. To be included, subjects had to be free of any acute or chronic pain; without recent use of analgesics, corticosteroids or medications with known effects on the CNS. Also, volunteers were not included if had abused of alcohol or psychotropic substances in the 6 months previous to the screening; and if had any rheumatologic, psychiatric, or neurological disorder. After obtaining written informed consent, a structured interview, and a blood sample was obtained. All biological samples were collected at the HCPA in agreement with institutional policies. None of the healthy volunteers underwent a thorough physical examination. Although apparently a healthy population might have an underlying disease, or asymptomatic tender points, the lack of pain symptoms, or the lack of analgesics or other drugs use in the last 6 months makes highly unlikely the presence of current disease, particularly during the cross-sectional evaluation of our study. None of the patients neither the pain-free volunteers received monetary or any other compensation for participating in the studies.

### Dependent and Independent Variables of Main Interest

The primary dependent variable was the measurement of the SICI and the change on the numerical pain scale (NPS 0–10) during a heterotopic stimulus. Secondary outcomes were other cortical excitability measures (MEP, SICF, and CSP). The independent variables of primary interest were the spectrum of structural pathology from persistent somatic nociception (i.e., OA) to absence of tissue injuries such as FM and MPS.

### Instruments and Assessments

(a) The motor cortex excitability was assessed using TMS with a MagPro X100 (MagVenture Company, Lucerne marked, Denmark) and a figure-8 coil. The coil was centered over the motor cortex (M1), held tangentially to the scalp to reach the midline at 45°. To ensure a relaxation of arms and correct positioning of the hand, subjects were sat in a comfortable reclining chair. Cortical excitability parameters were registered through surface electromyography recordings gathered at the contralateral right first dorsal interosseous muscles using Ag/AgCl electrodes. First, the resting motor threshold (RMT) was determined by obtaining five out of 10 consecutive trials MEPs with a peak-to-peak amplitude of 50 μV. Next, 10 MEPs were recorded with an intensity of 130% of the individual RMT. Moreover, the CSPs were assessed during muscle activity measured on a dynamometer set to approximately 20% of the maximal force. Accordingly, 10 CSPs were recorded using an intensity of 130% of the RMT. The SICI was assessed using an inter-stimulus interval of 2 ms. The conditioning stimulus (first) was set at 80% of the RMT while the test stimulus (second) was set at 100% of the individual MEP intensity. To assess the SICF was used an inter-stimulus interval of 12 ms. In total, 30 trials of paired-pulse were conducted in a randomized order (10 for each SICI, SICF, and control stimuli). We included the collection of all amplitudes of the MEPs, SICI and SICF and the duration of the CSPs in an off-line analyze. The units for these parameters included MEP in mV, SICI and SICF in their ratio to the MEP, and the CSP in milliseconds (Pascual-Leone et al., 1994).

(b) Quantitative sensory testing (QST) was used to assess heat pain thresholds using the method of limits with a computer Peltier-based device thermode (30 × 30 mm; Schestatsky et al., 2011). The thermode was attached to the skin on the ventral aspect of the mid-forearm, with an increase of temperature at a rate of 1°C/s, from 30°C to a maximum of 52°C. We asked the participants to press a button as soon as their felt mild pain (6/10) on the NPS ranging from 0 (no pain) to 10 (the worst pain). A single training session was offered before so participants could get familiar with the device. The thermode remained on the right ventral forearm, even though, it was slightly altered on trials to avoid either response suppression or sensitization of the cutaneous heat nociceptors. To evaluate the degree to which pain perception is modulated following the presentation of an initial heterotopic noxious stimulus (CPM), we used the QST during cold water immersion. This sensation was assessed raising the temperature to the point at which subjects felt mild pain (6/10) on the NPS. Thus, they immersed their non-dominant hands into cold water (zero to four degree Celsius) for 1 min. The QST was administered after 30 s of the cold-water immersion. During this time, subjects were asked to rate the pain of the stimulated arm (pain sensation by heat) using the same NPS. During the experiment for each participant, the temperature was held constant. The CPM was defined as the difference (presented in percentage) between the average pain rating before and after cold water immersion. To control for individual variation concerning baseline values, we used the proportion of difference from baseline.

### Potential Confounding Factors

The psychological tests used in the current study had been validated for the Brazilian population (Gomes-Oliveira et al., 2012; Sehn et al., 2012). Two independent medical examiners were trained to administer the pain scales and to conduct the psychological tests. The patients’ depressive symptoms were assessed using the Beck Depression Inventory II (Gomes-Oliveira et al., 2012). The catastrophizing thinking related to pain was evaluated using the Brazilian Portuguese of the Catastrophizing Scale (B-PCS; Sehn et al., 2012). We used a standardized questionnaire to assess demographic data and medical comorbidities.

### Serum Neuroplasticity Mediators’ Concentration

All of the trials used standard procedures for biological samples, by collecting blood at minimum 8 h after fasting early in the morning. All biological materials were collected before applying any intervention. Plastic tubes were centrifuged for 10 min at 5000 g at 4°C. Serum was frozen at −80°C until assays were performed. Serum neuroplasticity mediators concentrations were determined using specialized BDNF kits (catalog no. CYT306, the lower detection limit of the kit = 7.8 pg/mL, Chemicon/Millipore).

### Efforts to Address Potential Sources of Bias

To reduce assessment bias only two researchers (MGT; WC) with a practicing of the outpatient pain clinic at HCPA with vast clinical expertise were responsible for making the diagnostics according to pre-specified criteria. Three evaluators with specific training in performing TMS were responsible for all TMS measures of cortical excitability. The same evaluators applied clinical scales and performed the QST.

### Sample Size

The power of the study was estimated based on type II and type I error of 0.15 and 0.05 respectively and in anticipation of an effect size (*f*_2_ = determination coefficient) of 0.15 for the multiple hierarchical regression analysis allowing for four predictors (the *Post hoc* statistical power calculator for hierarchical multiple regression: 46^1^. A sample of 100 patients would detect an effect size for correlations of 0.15, with a power of 93% at a 0.05 alpha level.

### Statistical Analysis

To summarize the main characteristics of the sample we used traditional descriptive statistics, and performed Shapiro-Wilk tests to evaluate a normal distribution, we used Shapiro-Wilk tests. We used ANOVA to compare continuous variables with parametric distribution, and Chi-Square or Fisher’s exact test for categorical variables. Variables not normally distributed were log transformed for further inclusion into regression models.

To compare each cortical excitability parameter (MEP, CSP, SICI, SICF) among CPS (MPS, FM, OA) and healthy subjects we used ANOVA. While an MANCOVA was used to compare the relationship between the cortical excitability parameters as dependent variables (MEP, CSP, SICI, SICF) and the spectrum of structural pathology as a binomial independent variable (where, OA represents ongoing tissue injury and FM and MPS grouped together represent the absence of structural pathology). Another MANCOVA model was used to assess the relationship between the SICI and the CPM (dependent variables) with the BDNF, according to the spectrum of structural pathology. Taking into account that the pain severity, the age, the degree of depressive symptoms, and the use of psychotropic medications differed between the pain syndromes and that these factors can affect the biological process of BDNF secretion, we constructed an adjusted index. A multivariate regression model controlled by multicollinearity was used to obtain an adjusted index used as the surrogate of the BDNF. We adjusted for multiple comparisons using Bonferroni correction. Cohen’s f2 effect size was calculated using an effect size calculator for multiple regressions given the values of R2 [A-priori Sample Size Calculator for Hierarchical Multiple Regression^2^]. The data were analyzed using SPSS version 22.0 (SPSS, Chicago, IL, USA).

## Results

One hundred records were retrieved from three different trials ran in the HCPA. The sample of CS syndrome without structural pathology was composed of subjects with MPS (*n* = 54) and FM (*n* = 19). The sample of CS syndrome with persistent nociceptive input included subjects with OA (*n* = 27). The flow chart of this study is presented in Figure 1.

We screened 123 potential participants with a diagnosis of MPS, and 54 of them were included in the study. Subjects were excluded if they did not fulfill the criteria for MPS or due to the presence of another diagnosis (e.g., FM). We screened 38 potential participants with a diagnosis of FM, and 19 of them were included in the study. Subjects excluded did not fulfill the diagnostic criteria for FM or had other diagnoses (e.g., rheumatoid arthritis, chronic use of corticosteroids). We screened 85 potential participants with a diagnosis of OA, and 27 of them were included in the study. We excluded them for not fulfilling diagnostic criteria for severe OA, and due to the presence of other diagnoses (i.e., surgery in the segment, chronic use of corticosteroids, among others). We screened 27 HCs, and 14 were included in the study. They were excluded in the presence of depressive symptoms in moderate to severe intensity, history of epilepsy, chronic headache or use of psychotropics. Final sample characteristics are presented in Table 1.

**Table 1:** **Characteristics of the sample**.

Characteristic	Healthy subjects (*n* = 14)	Myofascial pain syndrome (*n* = 54)	Fibromyalgia (*n* = 19)	Osteoarthritis (*n* = 27)	*P*
Age (years)	32.43 (10.81)	46.13 (12.10)	50.42 (8.84)	64.42 (7.81)	0.00
Body mass index (kg/m^2^)	23.12 (2.93)	25.22 (4.37)	31.81 (7.08)	28.52 (5.52)	0.00
Years of education	17.14 (2.53)	10.16 (3.61)	13.29 (4.04)	10.37(5.61)	0.00
Employed (yes/no)	14/0	45/9	16/3	19/8	0.19
Smoking (yes/no)	0/14	2/52	5/14	0/27	0.14
History of psychiatric disorder (yes/no)*	NA	19/35	7/12	5/22	0.24
Drug active on the nervous system (yes/no)**	NA	17/37	11/8	12/15	0.11
Tricyclic antidepressant (yes/no)	NA	7/47	7/12	1/26	–
Selective serotonin reuptake inhibitor (yes/no)	NA	8/46	9/10	6/21	–
Anticonvulsants (yes/no)	NA	4/50	1/18	0/27	–
Benzodiazepine	NA	1/53	2/17	5/22	–
Chronic disease (yes/no)	NA	11/8	16/38	20/7	0.00
Hypertension (yes/no)	NA	14/40	10/9	14/13	–
Diabetes mellitus (yes/no)	NA	5/49	1/18	4/23	–
Asthma (yes/no)	NA	1/53	3/16	3/24	–
Number of analgesic doses used per week (≥4 doses-week/<4 doses)	NA	27/26	14/5	20/6	0.04
Pain on the VAS (last 7 days)	NA	7.23 (2.19)	7.94 (1.89)	6.26 (2.15)	0.03
Pain on the VAS (24 h)	NA	6.11 (2.59)	7.10 (1.88)	5.37 (2.47)	0.06
Beck depression inventory II	NA	13.92 (8.85)	24.47 (11.67)	10.04 (7.18)	0.00
Brazilian portuguese catastrophizing scale (B-PCS)	NA	28.26 (12.51)	34.68 (11.69)	22.89 (11.59)	0.00
Serum BDNF (ng/mL)	19.00 (8.79)	29.28 (20.01)	50.78 (16.06)	17.91 (7.27)	0.00

Data are presented as mean (standard deviation) or number of subjects (n = 114). *Patients could have none or more than one psychiatric disorder. **Some patients were using more than one type of drug. Abbreviations: visual analog scale for pain, from 0 to 10 (VAS); brain-derived neurotrophic factor (BDNF).

### Motor Cortex Excitability Parameters According to Chronic Pain Syndromes

The cortical excitability parameters are presented in Table 2. The mean (SD) between CPS and healthy subjects were compared using ANOVA followed by Bonferroni adjustment for multiple comparisons. Compared to healthy volunteers, subjects with MPS presented greater corticospinal tract excitability as shown by elevated MEPs. They also exhibited higher SICF but reduced SICI and CSP. As a matter of fact, MPS showed the largest MEPs amplitude among the subjects with CPS. Furthermore, the SICF was higher, while the SICI and CSP were lower in MPS compared to healthy volunteers. Except the MEP, the cortical excitability parameters of the MPS subjects were similar to those with FM, but different to those of OA subjects. Except for MEPs, FM subject’s cortical excitability was different to those of the HCs in the same direction as MPS were. Likewise, the cortical excitability parameters of the subjects with OA differed to the one of the HCs in the same direction as MPS and FM, which means having higher MEPs and SICF and lower SICI and CSP.

**Table 2:** **Cortical excitability parameters presented by chronic pain syndrome (CPS)**.

	Chronic pain syndrome			
	Fibromyalgia (*n* = 19)	Myofascial pain syndrome (*n* = 54)	Osteoarthritis (*n* = 27)	Healthy subjects (*n* = 14)
Motor evoked potential (mV)	1.13 (0.11)^a^	1.64 (0.49)^c^	1.46 (0.62)^b^	1.25 (0.38)^a^
Short intracortical facilitation (ratio: SICF/test stimulus)	0.96 (0.44)^c^	1.13 (0.23)^c^	0.97 (0.41)^c^	0.71 (0.36)^a^
Short interval intracortical inhibition (ratio: SICI/test stimulus)	0.32 (0.22)^c^	0.31 (0.17)^c^	0.59 (0.30)^b^	0.92 (0.07)^a^
Cortical silent period (CSP)	68.07 (18.43)^c^	68.44 (20.55)^c^	62.20 (16.68)^b,c^	76.67 (21.35)^a^

*Data are presented as mean (SD; n = 114). Different superscripts (a, b, c) indicate significant difference among treatment groups after post hoc analysis adjusted by Bonferroni (P < 0.05). Analysis of variance (ANOVA) to compare mean (SD)*.

### Cortical Excitability According to the Spectrum of Structural Pathology to Absence of Tissue Injury

The multivariate regression model with the cortical excitability parameters as dependent variables (MEP, SICF, SICI, CSP) using the spectrum of structural pathology to an absence of tissue injury as independent binomial variable, where structural pathology (i.e., OA) is one level, and the absence of tissue injury (MPS and FM pooled together) is the other level (please see Table 3). This analysis showed a significant relationship between the spectrum of structural pathology to an absence of tissue injury and SICI (Wilks’ *λ* = 0.93, *F* = 3.16 (2) = 85, *P* < 0.04). The power of this analysis was 0.80. Subjects with an absence of structural pathology presented greater disinhibition than those with persistent nociceptive input. The adjusted mean (SD) on the SICI observed in the absence of tissue injury was 56.36% lower than in those with persistent nociceptive input [0.31(0.18) vs. 0.55 (0.32)], respectively.

**Table 3:** **Relationship between motor cortex excitability according to the spectrum of structural pathology and absence of tissue injury (*n* = 100)**.

Dependent variable	Type III sum of squares	*df*	Mean square error	*F*	*P*	*Partial eta squared*
Motor-evoked-potential amplitude (mV)	2.10	2	1.05	4.11	0.02	0.09
Short intracortical facilitation (ratio: SICF/test stimulus)	0.19	2	0.09	0.86	0.42	0.02
Short interval intracortical inhibition (ratio: SICI/test stimulus) *log*	5.42	2	2.71	7.42	0.001	0.15
Cortical silent period (CSP)	2668.54	2	1334.27	3.55	0.03	0.08
	**B**		**SEM**	***t***	***P***	***Partial eta squared***
**Motor evoked potential (mV)**
Intercepted	2.06		0.37	5.64	0.000	0.27
Absence of structural pathology (*n* = 73)	−0.008		0.17	−0.05	0.96	0.00
Presence of structural pathology (*n* = 27)	0^a^		–	–	–	–
Age (years)	−0.01		0.005	−2.27	0.02	0.06
**Short intracortical facilitation (ratio: SICF/test stimulus)**
Intercepted	1.05		0.24	4.30	0.000	0.18
Absence of structural pathology (*n* = 73)	0.09		0.11	0.81	0.41	0.008
Presence of structural pathology (*n* = 27)	0^a^		–	–	–	–
Age (years)	−0.001		0.003	−0.30	0.76	0.001
**Short intracortical inhibition (ratio: SICI/test stimulus) log**
Intercepted	−0.79		0.43	−1.82	0.07	0.00.
Absence of structural pathology (*n = 73*)	−0.58		0.20	−2.84	0.006	0.09
Presence of structural pathology (*n* = 27)	0^a^		–	–	–	–
Age (years)	0.002		0.006	0.27	0.79	0.001
**Cortical silent period**
Intercepted	68.74		14.03	4.89	0.001	0.22
Absence of structural pathology (*n = 73*)	8.60		6.54	1.31	0.19	0.02
Presence of structural pathology (*n* = 27)	0^a^		–	–	–	–
Age (years)	−0.19		0.19	−0.99	0.32	0.01

### Relationship Between Intracortical Inhibition and Descendent Pain Modulatory System with The BDNF According to Structural Pathology

The lack of significant differences in cortical excitability parameters between MPS and FM supports the hypothesis that both pathologies do not differ significantly in their cortical facilitatory and inhibitory profile. Therefore, FM and MPS were grouped under the label of CSS with an absence of tissue injury for the subsequent analysis. As presented in Table 1, serum BDNF differs between healthy volunteers and subjects. When pooling subjects according to the presence of tissue injury, we observed that serum BDNF in those with the spectrum of structural pathology had significantly lower levels in comparison to those with absence of tissue injury, with 17.91 (7.27) and 35.29 (21.22), respectively (*t* = 3.830; *P* < 0.0001 of the comparison after log transformation of both serum BDNF). To account for the influence of pain severity, age, depressive symptoms, and use of psychotropic medications on the secretion of BDNF, an index was constructed using multivariate regression. The factors included explained 30% of the variance of the model.

The multivariate linear regression model included the SICI and the CPM as dependent variables, and the used as independent variables the structural pathology (absence vs. presence, binomial) and the BDNF adjusted index. The model is presented in Table 4. This analysis showed a significant relationship between the presence of structural pathology, the SICI and the CPM (Wilks’ *λ* = 0.90, *F* = 4.83, *P* < 0.01). The power of this analysis was 0.81. Subjects with an absence of structural pathology presented greater disinhibition than those with persistent nociceptive input. The increase in BDNF was associated with the lower efficiency of the descendent pain modulatory system. However, it was not observed any difference of the BDNF effect in the SICI when we compared it according to the presence of tissue injury. Thus, to address this important issue we conducted an additional regression analysis to assess the relationship between the SICI and BDNF despite the condition sustaining pain. This model revealed a *β* coefficient = −0.22; *t* = −2.14; *P* = 0.03, suggesting that the relationship between BDNF and ICI is independent of the chronic pain mechanism.

**Table 4:** **Relationship between intracortical inhibition and descendent pain modulatory system with the brain-derived neurotrophic factor (BDNF) according to the spectrum of structural pathology and absence of tissue injuries (*n* = 100)**.

Dependent variable	Type III sum of squares	*df*	Mean *square error*	*F*	*P*	*Partial eta* squared
**Change of NPS (0–10) during CPM-task**	73.097	3	24.36	4.60	0.005	0.30
**Short intracortical inhibition (*log*)**	3.88	3	1.29	3.75	0.014	0.11
	**B**		**SEM**	***t***	***P***	Partial eta *squared*
**Change of NPS (0–10) during CPM-task**
Intercepted	0.10		0.69	0.16	0.880	0.00
Absence of structural pathology (*n = 73*)	4.46		0.76	5.90	0.001	0.28
Presence of structural pathology (*n = 27*)	0^a^		–	–	–	–
BDNF (*adjusted index*)	−0.09		0.03	−3.02	0.003	0.09
**Interaction**
Absence of structural pathology *vs*. BDNF (*index adjusted; n = 65*)	−0.07		0.03	−2.31	0.020	0.06
Presence of structural pathology *vs*. BDNF (*index adjusted; n = 27*)	−0.29		0.07	−4.33	0.001	0.18
**Short intracortical inhibition (*log*)**
Intercepted	−0.80		0.18	−4.55	0.001	0.19
Absence of structural pathology (*n = 73*)	−0.45		0.19	−2.34	0.020	0.06
Presence of structural pathology (*n = 27*)	0^a^		–	–	–	–
BDNF (*index adjusted*)	0.00		0.008	0.05	0.95	0.00
**Interaction**
Absence of structural pathology *vs*. BDNF (*index adjusted; n = 65*)	−0.001		0.003	−0.29	0.78	0.001
Presence of structural pathology *vs*.BDNF (*index adjusted; n = 27*)^§^	0.01		0.006	1.77	0.08	0.04

*^§^Take in account that the severity of pain, age, depression symptoms, and use of psychotropic medication as dummy variable*.

**Figure 2:**
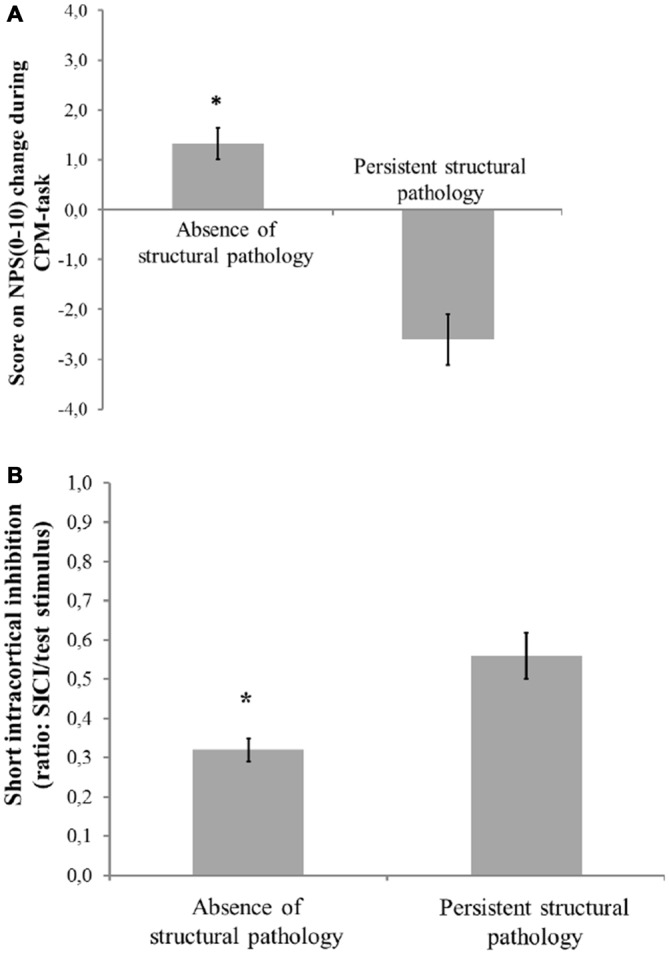
**Mean and standard error of mean (SEM). (A)** Change on numerical pain scale [NPS (0–10)] during conditioned pain modulation (CPM)-task; **(B)** Short intra-cortical inhibition (SICI) expresses the relationship between the amplitude of wave and motor evoked potentials (MEPs) (relative amplitude, express in %), at inter-stimuli intervals (ISIs) of 2 ms with paired-pulse. The first is a sub-threshold stimulus [80% of the rest motor threshold (rMT)] followed by the second one which is a suprathreshold stimulus (130% rMT). Spectrum of structural pathology to absence of tissue injury to persistent nociceptive input. The error bars indicate the SEM. An asterisk indicates a significant difference between groups according to spectrum of structural pathology to absence of tissue injury to persistent nociceptive (*P* < 0.05). A MANCOVA was used to perform the comparisons, followed by the Bonferroni correction for *post hoc* multiple comparisons.

Figures 2A,B presents the relationships between the SICI and the CPM (primary outcomes) according to the presence of structural pathology. The means were compared using MANCOVA, and *post hoc* were adjusted for multiple comparisons using Bonferroni correction (the model is presented in Table 4).

## Discussion

This study assessed the motor cortex excitability by using different TMS measures, such as MEP, CSP, SICI, SICF, and BDNF levels in chronic musculoskeletal pain according to structural pathology. These data suggest that there is a relationship between the motor cortex disinhibition and conditions of chronic musculoskeletal pain compared to healthy subjects. This disinhibition is greater in subjects with chronic pain without tissue injury compared to the ones with structural lesion (Table 4). Additionally, after adjusting for relevant confounders, higher levels of BDNF were significantly correlated with decreased inhibitory system as assessed by CPM.

We observed greater disinhibition at the cortical level when the CS syndrome occurred without evidence of structural pathology (i.e., MPS and FM) compared to those with persistent nociceptive input (i.e., OA). This finding suggests that a different activation of the nociceptive system leads to distinct plastic changes in the pain pathways. It is possible that this disinhibition process is a common feature of CPS, which could be further increased in the absence of structural pathology due to lack of opposition. Furthermore, the cortical inhibition could be used as an additional tool to infer, to a certain extent, the level of severity of the CS phenomena. This needs, however, further confirmation with large clinical trials.

This hypothesis is biologically plausible because the disinhibition results from the imbalance between excitability and inhibition induced by GABA activity reduction, an increase in glutamate activity, and activation of NMDA-dependent activity (Nitsche et al., 2010). These dysfunctions in excitatory/inhibitory systems at pain pathways are nothing but the biological grounds of the clinical picture known as CSS.

Our study suggests that the supraspinal reorganization in different chronic musculoskeletal pain conditions, which is in agreement with previous studies (Gracely et al., 2002; Flor, 2003). Despite being conceivable that due to the structural lesion, pain occurs by specific activation of pain pathways, a sustained activation of the nociceptive system leads to an involvement of different brain circuitries. While in the CS syndromes in the absence of an obvious source of nociception, a self-driven stimulus activates the pain circuits autonomously. Thereby, the present findings suggest the evidence for a disinhibition spectrum that was presented according to the pathophysiology of chronic musculoskeletal pain.

Our data also shows that the neuroplastic changes as assessed by the TMS measurements were related to both, the musculoskeletal CPS and its severity (interpreted as the gradient in evident structural pathology). An indirect evidence supporting these claims is that there are morphometric differences in prefrontal and thalamic gray matter between subjects with neuropathic and non-neuropathic chronic pain, which can suggest differential CNS neuroplasticity according to the etiology involved in the development of chronic pain (Apkarian et al., 2004). Also, corresponding neuroplastic changes were experimentally observed in a transient deafferentation-induced by an anesthetic nerve block (Theuvenet et al., 2011). Taken together, these findings suggest that the pathophysiological mechanisms and the severity of the disease moderate the disinhibition process. This hypothesis is also supported by further evidence in neuropathic pain, in which a more pronounced motor cortex disinhibition was observed in the moderate/severe pain (NRS ≥ 4) compared to the mild pain. The cortical reorganization has also been suggested following the use of rTMS applied to the motor cortex restored the equilibrium between the excitatory and inhibitory system in parallel to the reduction in pain intensity (Lefaucheur et al., 2006; Mhalla et al., 2011). Moreover, increased ICF and a decreased SICI in the contralateral hemisphere following limb amputation was shown (Chen et al., 1998; Schwenkreis et al., 2000), thus supporting a cortical reorganization. Likewise, subjects with complex regional pain syndrome (CRPS) presented a reduced SICI, not only in the ipsilateral but also in the contralateral motor cortex (Lenz et al., 2011). Similarly, SICI reduction was shown in several other different chronic pain conditions, such as MPS (Dall’Agnol et al., 2014), FM (Mhalla et al., 2010), OA (da Graca-Tarragó et al., 2015) and neuropathic pain syndromes (Schwenkreis et al., 2010).

The cortical excitability pattern of FM and MPS is not significantly different. The only difference is in the MEPs, which is thought to represent the corticospinal tract excitability (Petersen et al., 2003). In fact, these two entities might be the same syndrome, which represents a continuum at different moments at long of time. According to other authors have previously proposed such interpretation, although they offered scarce evidence supporting this point (Ge et al., 2009).

In the present study, the heterotopic nociceptive stimulus during the CPM-task induced a greater response in the descending modulatory system in subjects with persistent nociception compared to an absence of tissue injury. Although the mechanisms underlying this phenomenon are unknown, it is known that during inflammation the periaqueductal gray (PAG) suffers structural changes (Guan et al., 2002; Miki et al., 2002; Imbe et al., 2005). Furthermore, in the spinal cord, an increased turnover of noradrenaline (Weil-Fugazza et al., 1986) and the number of alpha(2)-adrenergic receptor has been associated with the inflammation (Brandt and Livingston, 1990). All these changes likely contribute to a rise in descending pain inhibition. It is possible that the inflammation contributes to triggering and maintenance of increased inhibitory controls. However, there are mixed results. Chronic arthritis induced experimentally strengthens the excitatory drive caused by conditioning stimulus (Danziger et al., 1999). Whereas in clinical studies subjects with FM presented a reduction of CPM, which potentially contributes to hyperalgesia (Kosek and Hansson, 1997) and in neuropathic pain the effect of CPM varied from a particular influence on on-going vs. evoked pain (Witting et al., 2003).

Although the mechanisms of facilitation induced by chronic pain prompt to disengagement in the descending modulation, according to the present findings this process can have a distinct level of severity according to the spectrum of structural pathology to an absence of tissue injury. The descendent modulation involves some mechanisms to inhibit the neurotransmission at the PAG, and at the spinal cord. These mechanisms include the activation of inhibitory of interneurons (Millar and Williams, 1989); reduction the quantity of amino acids, neuropeptides, and monoamines (Jensen and Yaksh, 1984); and postsynaptic inhibition of pain-relay neurons (Giesler et al., 1981). Additionally, the role of monoamines in order to increase the inhibition has already been demonstrated in preclinical studies, in which the duloxetine use reduces the amine uptake (Wong et al., 1993), and also in clinical research (for instance in knee OA), where the duloxetine decreased pain more than placebo (Chappell et al., 2009).

In this report, the reduced inhibition was inversely correlated with the BDNF despite the pain condition. This result highlights the remarkable effect of this neurotrophic factor in the cortical neuroplasticity process. Equally, the BDNF had an inverse correlation with the CPM, thus suggesting that decreased function in the descending pain modulatory system (greater scores in the CPM) prone to a higher propensity for pain. In this way, we showed that the variability of serum BDNF and the dynamic state of inter-hemispheric cortical excitability was independent of the mechanism underlying the chronic musculoskeletal pain. Thus, the correlation between the disinhibition in the motor cortex and the dysfunction of descending pain modulation system observed in this study might have clinical relevance because the CPM is a marker with a large size effect to identify impairment of descendent pain modulatory system in populations with long-term pain conditions (Lewis et al., 2012). Hence, it is biologically plausible that the BDNF enhancement activates signaling pathways in the spinothalamic tract due to a reduction of the GABAergic inhibitory effect (Spezia Adachi et al., 2015). Even so the design of this study prevents establishing if the disinhibition in the motor cortex could result in the higher serum levels of this neurotrophic factor, or vice-versa, it does permit us to a better comprehension of the dysfunctional disinhibition at cortical and intra-cortical regions in severe musculoskeletal chronic pain. Thereby, the pieces of evidence these findings possess may hold clinical implications such as to understand that the effects of BDNF in GABAergic/glycinergic system engage multiple molecular and cellular mechanisms that are largely complementary (i.e., increased excitation and reduced inhibition) in spinal, midbrain and peripheral structures associated with nociceptive processing. The secretion of BDNF by microglia downregulates of the Cl-cotransporter K^+^-Cl^−^ exporter (KCC2) expression in the dorsal horn resulting accumulation of intracellular chloride shifts the chloride equilibrium potential (ECl) to a less negative value. Hence, the activation of GABAA receptors produces a less hyperpolarization and a less inhibition (Prescott and De Koninck, 2005; Prescott et al., 2006). If ECl is sufficiently displaced, GABA may exert an excitatory effect and augments nociceptive transmission at the level of descending control mechanisms by a tyrosine receptor kinase B (TrkB)-dependent mechanism (Guo et al., 2006). In fact, there is compelling evidence that BDNF is a ubiquitous pain mediator at many levels of the nervous system. Given this, it is hard to conclude that the generation of BDNF is indeed a compensatory mechanism specifically associated with both, chronic inflammatory and neuropathic pain. So, these results support the hypothesis that the chronic pain induces reorganization in circuits involved in pain processing at cortical and in descending pain modulatory system. Also, they suggest that the pain thresholds are in opposite direction of BDNF level in severe chronic musculoskeletal pain independently of the pain mechanism.

However, several potential limitations in this study need to be addressed. From a biological perspective, the integration of cortical excitability, descendent modulatory system, and neuroplasticity makers has been showed in conditions other than pain, including inflammation, cancer, learning, memory, epilepsy, neurodegenerative, and neuropsychiatric disorders. Therefore, differences among different populations deserve additional consideration. Furthermore, a set of factors such as pain severity, age, analgesic and antidepressant use and depressive symptoms differed between our samples. As we demonstrated in a previous study, BDNF is a marker to distinguish to some extent the level of CS among several types of non-neuropathic chronic pain (Deitos et al., 2015). In previous studies, an association between BDNF levels and cortical excitability measures was shown (Kleim et al., 2006; Cheeran et al., 2008). In fact, in the clinical setting is not possible to assess directly and isolate the effect of each one of these potential confounding factors in the BDNF secretion, neither in the neurophysiological measures. Thus, to control for the potential concealed influence of these set of factors in the BDNF secretion and in the measures of cortical excitability we constructed an adjusted index. This approach allows us to evaluate the contribution of BDNF as the independent variable on the cortical excitability (SICI) and in the descending pain modulatory system function on a standard scale. To the best of our knowledge, the relationship between BDNF, cortical excitability, descending pain modulatory system and the mechanism of musculoskeletal pain syndrome have not been explored before. But the cross-sectional nature does not allow establishing a cause-effect relationship. Nonetheless, this is certainly a good starting point to generate hypotheses for future studies. Also, the present results should be carefully considered in male samples because BDNF levels seem to be sex-dependent (Stefani et al., 2012). Moreover, the chronic pain samples studied in this study do not (neither pretend) represent all cases of CS syndromes, such as neuropathic pain and disorders with little pain involvement (e.g., conditions with less structural involvement, such as hand OA).

The aim of this study, was to comprehend the changes associated with the CSS in the neurophysiological and neurobiological measures. Thus, from a musculoskeletal pain perspective, the MPS in initial stages may be triggered by peripheral nociceptor stimuli, which will induce changes in brain networks, which in turn will begin to generate self-inputs to sustain the pain sensation (Mense, 2010). Therefore, to reduce the heterogeneity in the sample with the absence of peripheral nociception, only subjects with MPS that had a neuropathic component and functional disability were initially included. Significant cortical alterations have already been demonstrated in this sample by our group, in which increased intracortical facilitation and a dysfunction in the descendent pain system was observed (Dall’Agnol et al., 2014). In fact, it is possible that the chronic pain syndrome with an absence of nociception tends to induce more disinhibition by the lack of contra-regulatory effects induced by the sustained nociception. Thus, from a clinical perspective, the classification of CSS according to the spectrum of tissue injuries provides a substrate for rehabilitation, because it was shown that CSS subjects with an absence of nociception current worst catastrophizing thinking related to pain (Soriano-Maldonado et al., 2015). Hence, therapeutic approaches could then also change maladaptive illness beliefs, and thus altering maladaptive pain cognitions. This can help in the clinical decision process, as well as helping in the construction of practical approaches for “unexplained” chronic musculoskeletal pain for both, clinical recognition (Nijs et al., 2010) and treatment (Nijs and Van Houdenhove, 2009; Nijs et al., 2010). Specifically, CSS therapies could target the neuroplasticity process using pharmacological (i.e., antidepressant, anticonvulsant, etc.) and non-pharmacological techniques such as transcranial direct current stimulation (tDCS), TMS, electro-acupuncture and other physical and cognitive therapies.

Also, in the present study, the serum level of BDNF can overestimate the central sources because we cannot isolate its source of other structures besides the brain. In this way, a recent study showed BDNF gene in primary cultures of megakaryocytes of rats and human, which suggests that the platelets could represent the largest source of BDNF (Chacón-Fernández et al., 2016). In spite of this; it has also been demonstrated, that the circulating BDNF represents 70–80% of the one produced in the CNS (Rasmussen et al., 2009). Also, an experimental study in rats showed a correlation of about 0.8 between the serum levels of BDNF and its concentration in the cerebral cortex (Karege et al., 2002). Although the transport of BDNF produced in the CNS occurs through the blood-brain barrier (BBB) via saturable systems, this data suggests that the fluctuations of this neurotrophin in the blood reflect changes in the nervous system (Poduslo and Curran, 1996; Asmundson et al., 1999). Furthermore, there are a significant number of studies demonstrating that occur variations in the serum levels of BDNF after interventions with effect in the CNS (Okamoto et al., 2008; Solati et al., 2015; Jeong et al., 2016; Kawazu et al., 2016; Niimi et al., 2016; Wens et al., 2016). These approaches therapeutics include antidepressant drugs (Brunoni et al., 2008), electroconvulsive therapy (ECT; Brunoni et al., 2014), TMS (Dall’Agnol et al., 2014) and tDCS (Brietzke et al., 2015). Haile et al. (2014) demonstrated similar results post-infusion of ketamine, where they observed a higher increase in serum levels in responders compared to non-responders. Thus, this set of evidence shows that changes in peripheral BDNF levels are associated with clinical outcome involving a neuroplasticity process, and they suggest that at least part of BDNF is produced in CNS. Nevertheless, we should have parsimony in the interpretation these results because we can infer only indirectly changes of BDNF from the brain.

In sum, the present findings showed greater disinhibition in the motor cortex and the descending inhibitory pain modulation system in FM and MPS than in OA. Likewise, the inter-hemispheric disinhibition as well as the dysfunction in the descending pain modulatory system is higher in chronic pain with the absence of tissue injury compared to chronic pain with a structural lesion. Finally, increased level of serum BDNF mediated the disinhibition of motor cortex excitability, as well as the function of descending inhibitory pain modulation system, independently of the physiopathology mechanism involved in these musculoskeletal pain syndromes.

## Author Contributions

AD: participated in the sequence alignment, participated in the design of the study, drafted the manuscript and approved the final version to be published. FC: participated in the sequence alignment, participated in the design of the study and approved the final version to be published. JAD-S: participated in the sequence alignment, participated in the design of the study, drafted the manuscript and approved the final version to be published. MdG and LT: participated in the sequence alignment, participated in the design of the study and approved the final version to be published. AS: participated in the sequence alignment and approved the final version to be published. ILdST: drafted the manuscript and approved the final version to be published. SC: drafted the manuscript and approved the final version to be published. JL: drafted the manuscript and approved the final version to be published. FF: drafted the manuscript and approved the final version to be published. WC: conceived the study, performed the statistical analysis, participated in the design of the study, drafted the manuscript and approved the final version to be published.

## Further Information

Registration in ClinicalTrials.gov: FM NCT01904097; MPS NCT01964729; OA NCT01747070.

## Conflict of Interest Statement

The authors declare that the research was conducted in the absence of any commercial or financial relationships that could be construed as a potential conflict of interest. The reviewer RB and handling Editor declared their shared affiliation, and the handling Editor states that the process nevertheless met the standards of a fair and objective review.

## Footnotes

^1^http://www.danielsoper.com/statcalc3/calc.aspx?id

^2^http://www.danielsoper.com/statcalc
